# Leukotriene D_4_-induced Caco-2 cell proliferation is mediated by prostaglandin E_2_ synthesis

**DOI:** 10.14814/phy2.12417

**Published:** 2015-07-26

**Authors:** Marisol Cabral, Raquel Martín-Venegas, Juan J Moreno

**Affiliations:** Departament de Fisiologia, Facultat de Farmàcia, Universitat de BarcelonaBarcelona, Spain

**Keywords:** 5-lipoxygenase, arachidonic acid cascade, cell cycle, cell growth, colon cancer

## Abstract

Leukotriene D_4_ (LTD_4_) is a pro-inflammatory mediator formed from arachidonic acid through the action of 5-lipoxygenase (5-LOX). Its biological effects are mediated by at least two G-coupled plasmatic cysteinyl LT receptors (CysLT_1-2_R). It has been reported an upregulation of the 5-LOX pathway in tumor tissue unlike in normal colon mucosa. Colon tumors generally have an increased expression of CysLT_1_R and colon cancer patients with high expression levels of CysLT_1_R have poor prognosis. We previously observed that the cyclooxygenase pathway is involved in the control of intestinal epithelial cancer cell growth through PGE_2_ production. The aim of this study was therefore to assess the effect of LTD_4_ binding with CysLT_1_R on Caco-2 cell growth. We note a number of key findings from this research. We observed that at a concentration similar to that found under inflammatory conditions, LTD_4_ was able to induce Caco-2 cell proliferation and DNA synthesis. Moreover, with the use of a specific receptor antagonist this study has demonstrated that the effect of LTD_4_ is a result of its interaction with CystLT_1_R. We also note the possible participation of the PLC-IP_3_-Ca^2+^/DAG-PKC signaling pathways in cytosolic PLA_2_ and [^3^H]AA release induced by LTD_4_-CystLT_1_R interaction. Finally, we found that the resulting activation of the AA cascade and the production of PGE_2_ eicosanoid could be related to the activation of cell signaling pathways such as ERK and CREB. These findings will help facilitate our understanding of how inflammatory mediators can affect the survival and dissemination of intestinal carcinoma cells.

## Introduction

Leukotriene D_4_ (LTD_4_) is a powerful pro-inflammatory mediator, which is formed from arachidonic acid through the action of 5-lipoxygenase (5-LOX) (Samuelsson 1979). LTD_4_ mediates its effects through specific cell surface receptors that belong to the G protein-coupled receptor family, cysteinyl leukotriene receptors (CysLTR). Two such receptors have previously been cloned: CysLT_1_R (Lynch et al. 1999) and CysLT_2_R (Heise et al. 2000). CysLT_1_R has the highest affinity of the two receptors (Heise et al. 2000). As such it has a higher affinity for LTD_4_ than CysLT_2_R (Lynch et al. 1999).

LTD_4_ is associated with the pathogenesis of several inflammatory disorders such as inflammatory bowel disease (IBD) (Stenton 1990). Not only is there a well-established connection between IBD and increased frequency of neoplastic transformation (Smalley and Dubois 1997), but a more general link between chronic inflammation and an increased risk of developing cancer has been suggested in previous studies (Coussens and Werb 2002). A cause-and-effect link has been established between chronic inflammation and colon cancer, which occurs via the activation and over-expression of the enzymes 5-LOX and cyclooxygenase-2 (COX-2). These enzymes are responsible for regulating the production of LTs and prostaglandins (PGs), respectively (Coussens and Werb 2002; Qiao and Li 2014). Unlike non-transformed human epithelial cells, CysLT_2_R is downregulated in the colon cancer cell lines (Magnusson et al. 2007). In contrast, it has been demonstrated that CysLT_1_R is upregulated in colon cancer tissue and that the binding of LTD_4_ to this receptor facilitates the survival of the cells in this tissue and negatively correlates with patient survival (Öhd et al. 2000, 2003). In accordance with this trend, Magnusson et al. (2007, 2010) recently observed that colon cancer patients with high expression levels of CysLT_1_R exhibited a poor prognosis. Moreover, as noted by Yudina et al. (2008), LTD_4_ upregulates 5-LOX, COX-2, and CysLT_1_R levels in intestinal epithelial cells providing a mechanism for maintaining inflammation and tumor progression.

In our study, we observed that through CysLT_1_R binding, LTD_4_ increases the release of arachidonic acid (AA) and the synthesis of PGE_2_. In addition, we found that this prostaglandin is responsible for the proliferative effects induced by LTD_4_ on intestinal epithelial Caco-2 cells.

## Materials and Methods

### Materials

LTD_4_, PGE_2_ and murine COX-2 were purchased from Cayman Chemical (Ann Arbor, MI). Non-essential amino acids, FBS, BSA, Fura-2 acetoxymethylester (Fura-2 AM), U73122, dantrolene, Gö 6983, ketoprofen, LY 171883, MK 571, NS 398, SC560 and SC19220, PD98059, ethidium bromide and acridine orange were purchased from Sigma Chemical (St. Louis, MO). LY 255283 was purchased from Tocris Biosc. (Bristol, UK). Arachidonyl trifluoromethylketone (AACOCF_3_) and bromoenol lactone (BEL) were acquired from Alexis Corp. (San Diego, CA). [Methyl-^3^H]thymidine (20 Ci/mmol) and [5,6,8,9,11,12,-14,15-^3^H] arachidonic acid ([^3^H]AA) (60-100 Ci/mmol) were from American Radiolabeled Chemicals Inc. (St. Louis, MO), and AH 23838 was kindly provided by Glaxo-Wellcome (Stevenage, UK).

### Cell culture and cell growth assay

Caco-2 cells, derived from a colon adenocarcinoma, were provided by the American Type Culture Collection (HTB-37, Manassas, VA). The cells were routinely grown in plastic flasks at a density of 10^4^ cells/cm^2^ and cultured in DMEM supplemented with 4.5 g/L D-glucose, 1% (v/v) nonessential amino acids, 2 mmol/L L-glutamine, 10% (v/v) heat-inactivated FBS, 100 U/mL penicillin and 0.1 mg/mL streptomycin at 37°C in a modified atmosphere of 5% CO_2_ in air, as previously described (Martín-Venegas et al. 2006). The growth medium was replaced twice per week and the day before the experiment. All the experiments were performed in pre-confluent cultures and consequently, in nondifferentiated cells. Caco-2 cell differentiation began when they reached the confluence and finished after 2 weeks postconfluence, following a previously described process (Martín-Venegas et al. 2006).

To perform the cell growth assay, cells were harvested with trypsin/EDTA and passed to 12 mm plastic clusters at a density of 10^4^ cells/cm^2^. After 4 days in culture, cells were incubated with treatments for a period of 48 h. Then, cell density was around 40 and 80 × 10^3^ cells/cm^2^ in absence or presence of FBS, respectively. Consequently all experiments were performed before reaching cell confluence. Cells were then washed, trypsinized, and counted with a microscope using ethidium bromide/acridine orange staining to view the number of viable cells (Parks et al. 1979).

### Analysis of DNA synthesis

DNA synthesis was measured using a [^3^H]thymidine incorporation assay. Caco-2 cell cultures were kept on 24-well plates in DMEM with 10% FBS at a density of 10^4^ cells/cm^2^. After 4 days in culture, the cells were incubated for 48 h with the treatments; [^3^H]thymidine (0.1 μCi/well) was added for the last 24 h. The media containing [^3^H]thymidine were then aspirated and cells were washed, overlaid with 1% Triton X-100 and scraped off the wells (Cabral et al. 2013). Finally, radioactivity present in the cell fraction was measured by scintillation counting, using a Packard Tri-Carb 1500 counter (Downers Grove, IL).

### Prostaglandin E_2_ (PGE_2_) analysis by enzyme immunoassay

PGE_2_ determination was performed using a competitive EIA kit (Cayman, Ann Arbor, MI) following the manufacturer’s instructions. Briefly, following a previously described process cells were maintained in 12 mm plastic clusters at a density of 10^4^ cells/well (Cabral et al. 2013). After 4 days in culture, Caco-2 cells were incubated for 60 min at 37°C with LTD_4_ (10 nmol/L) in the absence or presence of CysLT_1_R antagonists or a COX inhibitor. Finally supernatants were harvested and PGE_2_ was determined.

### Incorporation and release of [^3^H]AA

Cells were harvested with trypsin/EDTA and passed to 24-well plates at a density of 10^4^ cells/cm^2^. After 4 days, cells were FBS starvated during 24 h and then the medium was replaced by 0.5 mL DMEM containing 0.1% fatty acid free BSA and 0.1 μCi [^3^H]AA (1 nmol/L) for a period of 6 h. Cells were then washed three times with 0.5% BSA-containing medium to remove any unincorporated [^3^H]AA. After the study period (2 h), the medium was removed to determine the amount of [^3^H] radioactivity release. The amount of [^3^H]AA released into the medium was expressed as a percentage of cell-incorporated [^3^H]AA, which was determined in solubilized cells, as previously described (Martín-Venegas et al. 2006).

### Western blot analysis

Cells were seeded in 60 mm plastic clusters (10^4^ cells/cm^2^) and after 4 days the cultures were washed twice with ice-cold PBS, scraped off into PBS containing 2 mmol/L sodium EDTA and pelleted. These pellets were sonicated in PBS containing 4 mmol/L sodium EDTA, 500 μg/mL aprotinin, 500 μg/mL leupeptin, 500 μg/mL PMSF, and 400 μg/mL diethyldithiocarbamic acid, then resuspended in a lysis buffer containing 200 mmol/L Tris-HCl, 200 mmol/L NaCl, 2% Igepal CA-630 and 200 μmol/L DTT. Finally, an immunoblot analysis for COX-2 was performed, as previously described (Martín-Venegas et al. 2006). For β-actin immunoblotting, the monoclonal actin antibody (1:500) was used (Santa Cruz, Dallas, TX).

### Measurement of the cell signaling activated by eicosanoids

Cells were seeded in 60 mm plastic clusters (10^4^ cells/cm^2^) and after 4 days the cultures were incubated with the treatments (5 or 15 min) as previously described (Cabral et al. 2013). To measure the kinase activity with total cell lysates, Caco-2 cells were lysed using a denaturing cell lysis buffer containing 6 mol/L urea and protease (leupeptin 2 μg/mL, pepstatin 10 μmol/L, aprotinin 3 μg/mL) and phosphatase (NaF 5 mmol/L, Na_4_P_2_O_7_ 2 mmol/L, Na_3_VO_4_ 1 mmol/L) inhibitors. The resulting solutions containing 80–100 μg of proteins were then added to kinase ELISA plate and the assay was performed, following the manufacturer’s instructions (Symansis, Auckland, New Zealand). Optical density was then measured at 450 nm using a TECAN absorbance reader (Tecan Austria Gmbh, Salzburg, Austria). This simultaneous assay for the activation of multiple kinases provides a qualitatively better alternative to western blotting. We studied the effect of eicosanoids on the phosphorylation of Akt1 (pS473), Akt2 (pS474), ERK1/2 (pT202/Y204; pT185/Y187), GSK3β (pS9), p38α (pT180/Y182) and CREB (pS133) on the dephosphorylation of β-catenin (DP S33/S37/S41). The phosphorylation of Akt, ERK and p38 was measured after 5 min incubation with LTD_4_, whereas the phosphorylation of CREB, GSK and the dephosphorylation of β-catenin was assayed after 15 min.

### Statistical analysis

Results are expressed as mean ± SEM. All data were compared by one-way ANOVA and Student’s *t*-test using SPSS software (SPSS Inc., Chicago, IL). *P* < 0.05 was considered to denote significance.

## Results

**Figure 1:**
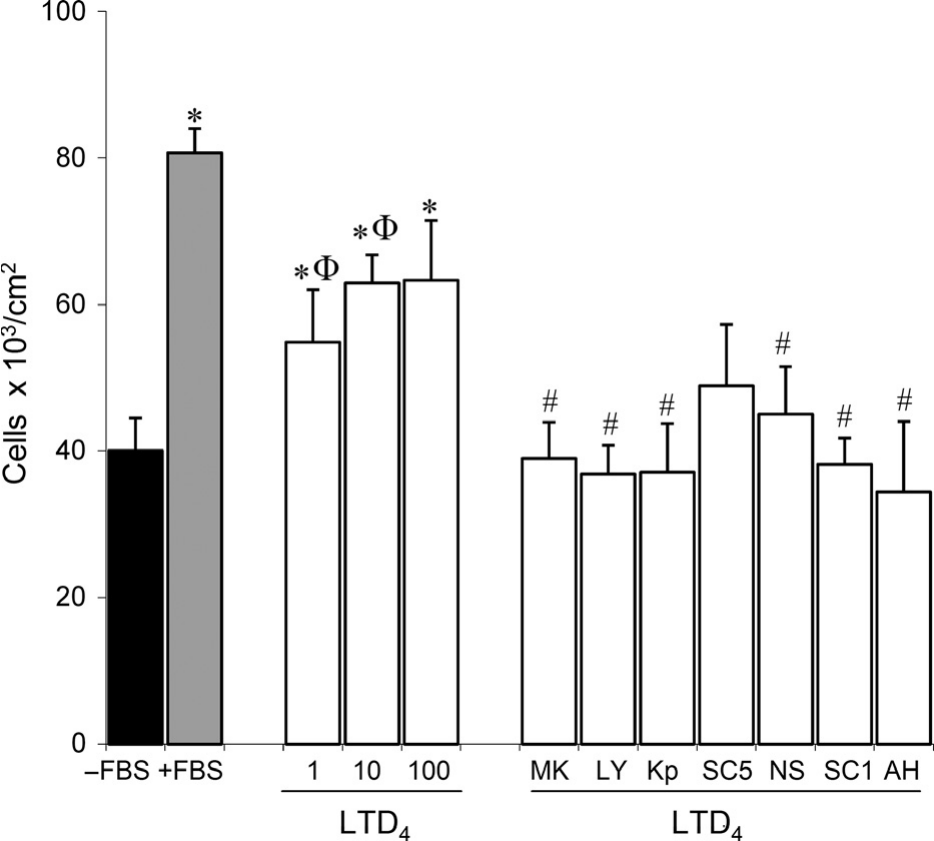
The effect of Leukotriene D_4_ (LTD_4_) on Caco-2 cell growth. Cells were incubated for 48 h in the presence of LD_4_ (1–100 nmol/L) and in the absence of growth factors (FBS); cells were then counted. Cell growth in control conditions (in the absence or presence of FBS) was included. LTD_4_ (10 nmol/L) was incubated in the presence of MK 571 (MK5, 25 μmol/L), LY171883 (LY1, 25 μmol/L), ketoprofen (Kp, 5 μmol/L), SC 560 (SC5, 60 nmol/L), NS 398 (NS, 5 μmol/L), SC 19220 (SC1, 60 nmol/L) or AH 23838 (AH, 20 nmol/L). Results are expressed as mean ± SEM of 4-5 determinations performed in triplicate. *P < 0.05 versus Caco-2 cells cultured without FBS; ^ϕ^*P* < 0.05 versus Caco-2 cells cultured with FBS; ^#^*P* < 0.05 versus Caco-2 cells cultured with LTD_4_ (10 nmol/L).

Figure1 shows that LTD_4_ (1–100 nmol/L) increases the number of viable cells in Caco-2 cell cultures in comparison with the results obtained in the absence of any growth factor. We note that the effect induced by LTD_4_ (10 nmol/L) was reduced in the presence of CysLT_1_R antagonists (MK 571 and LY 171883), a COX inhibitor (ketoprofen), a specific COX-2 inhibitor (NS 398), and EP_1_ or EP_4_ antagonists (SC 19220 and AH 23848, respectively) (Fig.1), whereas the effect of specific COX-1 inhibitor (SC 560) did not reach significance. The mentioned treatments did not cause cell detachment nor a decrease in cell viability at the concentrations tested, as confirmed by microscopic observation (data not shown).

**Figure 2:**
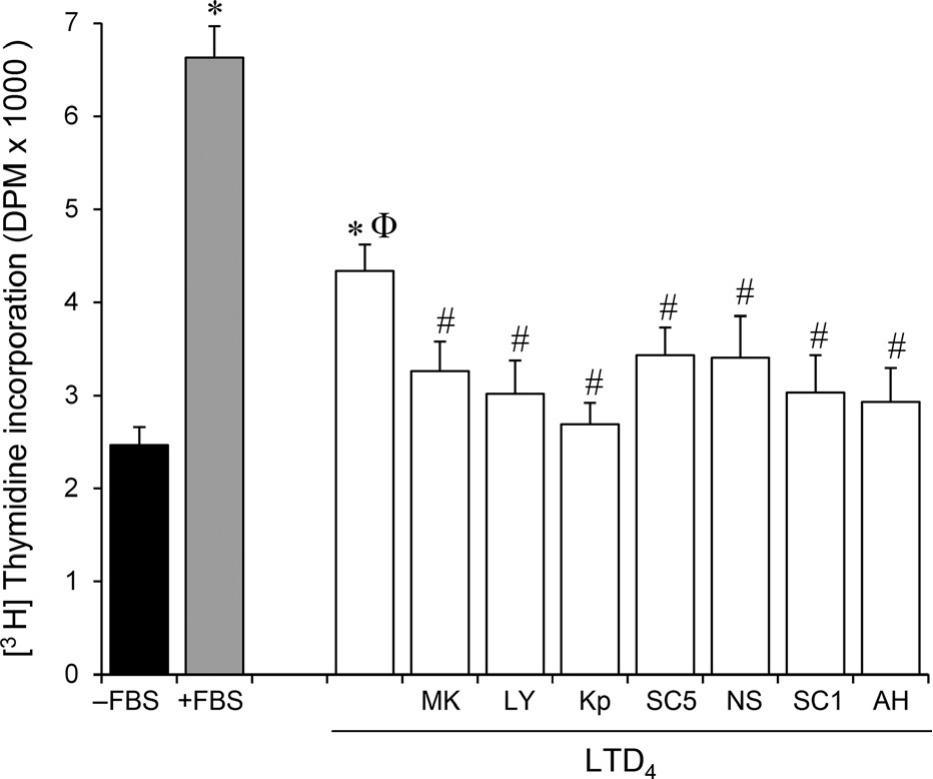
The effect of Leukotriene D_4_ (LTD_4_) on DNA synthesis. Caco-2 cells were incubated for 48 h in the presence of LTD_4_ (10 nmol/L) and in the absence of growth factors (FBS); [^3^H]thymidine incorporation into cells was then measured. DNA synthesis in control conditions (in the absence or presence of FBS) was included. LTD_4_ (10 nmol/L) was incubated in the presence of MK 571 (MK5, 25 μmol/L), LY171883 (LY1, 25 μmol/L), ketoprofen (Kp, 5 μmol/L), SC 560 (SC5, 60 nmol/L), NS 398 (NS, 5 μmol/L), SC 19220 (SC1, 60 nmol/L) or AH 23838 (AH, 20 nmol/L). Results are expressed as mean ± SEM of 4–5 determinations performed in triplicate. *P < 0.05 versus Caco-2 cells cultured without FBS; ^ϕ^*P* < 0.05 versus Caco-2 cells cultured with FBS; ^#^*P* < 0.05 versus Caco-2 cells cultured with LTD_4_.

The mitogenic effect observed with LTD_4_ was confirmed using [^3^H]thymidine incorporation (Fig.2). We found that LTD_4_ (10 nmol/L) induced DNA synthesis, whereas the above mentioned CysLT_1_R antagonists (i.e., MK 571 and LY 171883), the COX inhibitors, and the EP antagonists significantly inhibited the incorporation of [^3^H]thymidine into Caco-2 cells induced by LTD_4_.

**Figure 3:**
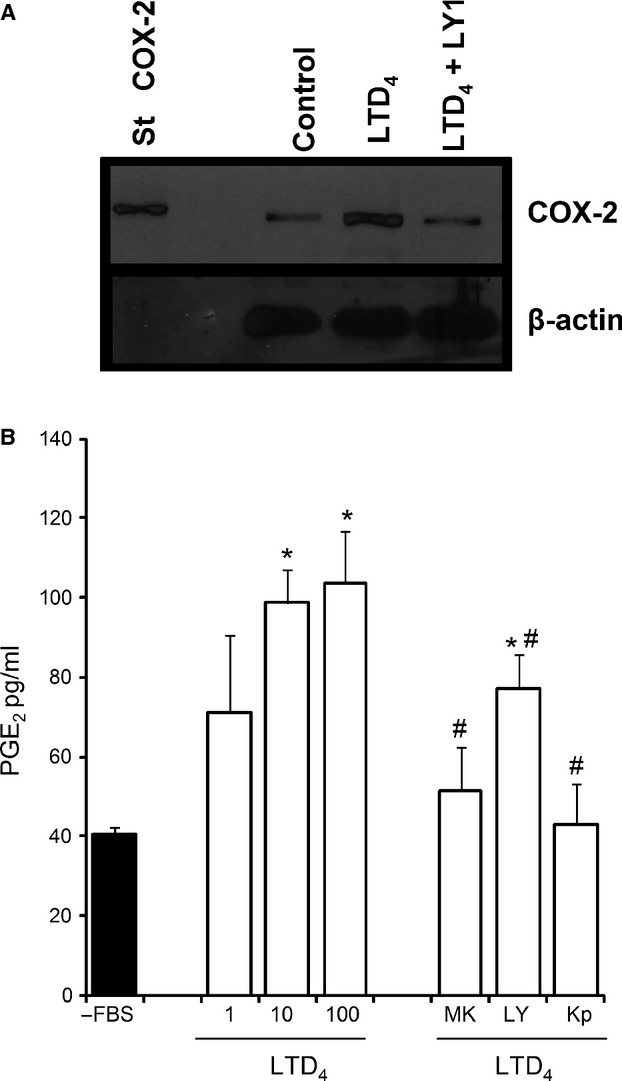
The effect of Leukotriene D_4_ (LTD_4_) on COX-2 expression and PGE_2_ synthesis. (A) Cells were incubated in the absence of FBS (Control) and in the presence of LTD_4_ (10 nmol/L) or LTD_4_ plus LY171883 (LY1, 25 μmol/L) for 30 min and COX-2 expression was determined using a specific antibody. Murine COX-2 (20 ng) was used as standard (St COX-2). Western blot was used in three experiments. (B) Caco-2 cells were incubated with LTD_4_ (1–100 nmol/L) for 15 min and PGE_2_ synthesis was determined. PGE_2_ synthesis induced by LTD_4_ (10 nmol/L) was studied in the presence of MK 571 (MK5, 25 μmol/L), LY171883 (LY1, 25 μmol/L) or ketoprofen (Kp, 5 μmol/L). Results are expressed in mean ± SEM of 5 determinations performed in triplicate. *P <* *0.05 versus Caco-2 cell cultures in the absence of growth factors (FBS); ^#^*P *<* *0.05 versus cells incubated with LTD_4_ (10 nmol/L).

LTD_4_ (10 nmol/L) was also able to induce a significant release of [^3^H]AA by Caco-2 cells, which was blocked in the presence of CystLT_1_R antagonists (MK 571 and LY 171883) (Table1). Moreover, the release of [^3^H]AA induced by LTD_4_ was blocked by a number of inhibitors: a PLC inhibitor (U 73122); an inhibitor that prevents the release of calcium from the endoplasmic reticulum (dantrolene), and by a PKC inhibitor (Gö 6983). We observed that the release of [^3^H]AA induced by LTD_4_ was also inhibited by a nonspecific phospholipase A_2_ (PLA_2_) inhibitor (AACOCF_3_) but not by a specific calcium-independent PLA_2_ inhibitor, for example, BEL. In addition, our study showed that LTD_4_ (10 nmol/L) increases the expression of COX-2, and that this effect was reverted by a CysLT_1_R antagonist (Fig.3A). In addition, we found that LTD_4_ induced the synthesis of PGE_2_ and that this action was reverted by CystLT_1_R antagonists (MK 571 and LY 171883) and ketoprofen (Fig.3B).

**Table 1:** [^3^H]AA release induced by leukotriene D_4_ (LTD_4_)

	[^3^H]AA (%)
Control	4.1 ± 0.3
LTD_4_	23.6 ± 1.8*
LTD_4_ + AACOCF_3_ (10 μmol/L)	8.2 ± 0.7*,#
LTD_4_ + BEL (10 μmol/L)	21.3 ± 1.7*
LTD_4_ + MK 571 (25 μmol/L)	7.2 ± 1.1#
LTD_4_ + LY 171883 (25 μmol/L)	7.9 ± 1.5#
LTD_4_ + U 73122 (0.1 μmol/L)	11.5 ± 2.1*,#
LTD_4_ + Dantrolene (50 μmol/L)	12.6 ± 1.8*,#
LTD_4_ + Gö 6983 (1 μmol/L)	13.4 ± 1.3*,#

[^3^H]AA was determined in non-differentiated Caco-2 cell cultures, as described in the Materials and Methods section. Cells were incubated in the presence of LTD_4_ (10 nmol/L) or LTD_4_ plus treatments and [^3^H]AA was determined 30 min after LTD_4_ incubation, respectively. Values are means ± SEM of three experiments performed in triplicate

* *P* < 0.05 versus control

# *P* < 0.05 versus LTD_4_.

**Figure 4:**
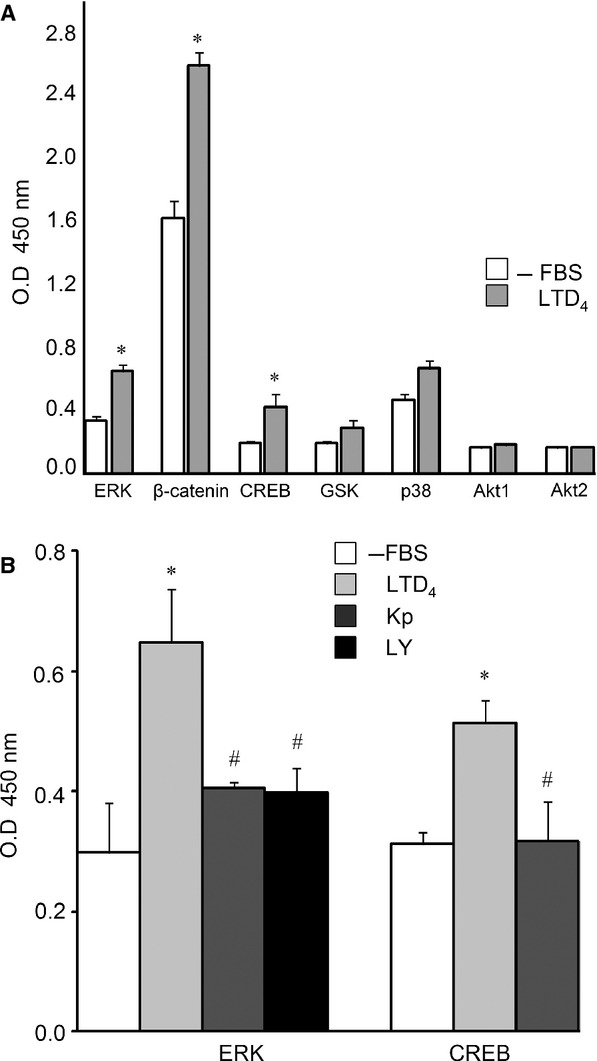
The effect of Leukotriene D_4_ (LTD_4_) on cell signaling. (A) Caco-2 cells were incubated with LTD_4_ (10 nmol/L) for 5 or 15 min and then cells were collected. Phosphorylated Akt1, Akt2, ERK1/2, p38α, CREB, and GSKβ and dephosphorylated β-catenin were then measured, as described in the Material and Methods section. (B) The effect of LTD_4_ (10 nmol/L) on ERK1/2 and CREB pathways. Cells were incubated with LTD_4_ (10 nmol/L) for 5 min in the presence of LY171883 (LY1, 25 μmol/L) or ketoprofen (Kp, 5 μmol/L). Values are mean ± SEM of triplicate determinations, the experiment was repeated twice. *P < 0.05 versus Caco-2 cells cultures in the absence of LTD_4_ (10 nmol/L); ^#^*P *<* *0.05 versus cells incubated with LTD_4_ (10 nmol/L).

Finally, we studied the capacity of LTD_4_ to phosphorylate pivotal elements in the cell signaling pathways implicated in the regulation of cell growth. For ERK, phosphorylation was highest after 5 min incubation and for CREB after 15 min. Dephosphorylation of β-catenin also increased after 15 min incubation (Fig.4A). In addition, our results show that ERK phosphorylation induced by LTD_4_ was reverted by a CysLT_1_R antagonist as well as by a COX inhibitor (Fig.4B) and that CREB phosphorylation induced by LTD_4_ was also blocked by ketoprofen (Fig.4B).

## Discussion

Colorectal cancer is the third most common cancer in the Western world and almost half of patients die of metastatic disease. This highlights the importance of research into the molecular mechanisms involved and their role in prognosis. Previous findings in our laboratory have demonstrated that the release of AA by PLA_2_s participates in the signaling pathways involved in the control of intestinal epithelial cell proliferation (Sanchez and Moreno 2002a), and that its subsequent metabolism by COX-2 could be involved in the control of Caco-2 cell growth. Research has shown that in tumor tissue, COX and 5-LOX pathways are upregulated, which is not seen in normal colon mucosa (Cianchi et al. 2006). Moreover, as Cianchi et al. (2006) reported, 5-LOX inhibition increases the antitumor activity of COX inhibitors in human colon cancer cells. These findings support the hypothesis that the key elements of the AA cascade are involved in the regulation of intestinal epithelial structure/function (Ferrer and Moreno 2010).

Recently, we reported that pre-confluent Caco-2 cells were able to synthesize LTB_4_ and 5-, and 12- and 15-HETE eicosanoids, which were found to be involved in the regulation of Caco-2 cell growth (Cabral et al. 2013). Dreyling et al. (1986) reported that human gastrointestinal tissues could synthesize cysteinyl leukotrienes, however, we were unable to detect LTD_4_ in Caco-2 cell culture supernatants (Cabral et al. 2013). Paruchuri et al. (2006) reported that cysteinyl LTs released from Caco-2 cells reached a concentration of 5 pmol/L, which is notably lower than the limits of detection (0.3 mmol/L) for this eicosanoid in our experimental conditions (Martín-Venegas et al. 2011).

It is important to consider that the tumor microenvironment has often been associated with infiltrating leukocytes in the tumor tissue and the surrounding stroma (Negus et al. 1997). Consequently, the activation of macrophages and mast cells in inflammatory processes and cancer might induce an additional release of cysteinyl LTs, such as LTD_4_, in the intestinal mucosa. Thus, our findings demonstrate that at a concentration range of 1–100 nmol/L, which is a level likely reached under tumorigenic conditions, LTD_4_ can induce Caco-2 cell proliferation and DNA synthesis. Moreover, our results have confirmed, using specific receptorial antagonists, that this effect is a consequence of the interaction with CystLT_1_R.

This effect was also reported by Magnusson et al. (2007), who demonstrated that unlike CysLT_2_R, CysLT_1_R is involved in intestinal epithelial proliferation. Moreover, it has been described that this receptor is upregulated in colon cancer and correlates with a poorer prognosis (Öhd et al. 2003; Magnusson et al. 2010). Similarly, we recently reported that the LTD_4_-CystLT_1_R interaction increases intracellular Ca^2+^ concentrations in Caco-2 cells, indicating that PLC activation as well as stores of extracellular Ca^2+^ and intracellular Ca^2+^ are involved in this event (Rodríguez-Lagunas et al. 2013). Our results indicate that the PLC-IP_3_-Ca^2+^/DAG-PKC signaling pathways and cytosolic PLA_2_ participate in the release of [^3^H]AA induced by the LTD_4_-CystLT_1_R interaction. Consequently this also demonstrates their participation in the activation of the AA cascade and eicosanoid production. These findings are consistent with Parhamifar et al. (2005) who reported that cytosolic PLA_2_ was activated and translocated to the nucleus upon LTD_4_ stimulation via a Ca^2+^-dependent mechanism that involves the activation of PKC in Caco-2 cells.

Furthermore, we observed that the interaction of LTD_4_ with the CystL_1_T receptor stimulated COX-2 expression, which is consistent with previous research, carried out by Yudina et al. (2008) using different intestinal epithelial cells. We can therefore surmise that PGE_2_ synthesis was induced by LTD_4_, which is consistent with the results obtained by Öhd et al. (2000) and Massoumi et al. (2003). Given that Caco-2 cell proliferation induced by LTD_4_ was reverted by nonspecific and specific COX inhibitors as well as EP_1_ and EP_4_ antagonists, we propose that this action is completely dependent of PGE_2_ synthesized by both COX as well as by PGE_2_ interaction with EP_1_ and EP_4_ that stimulated cell signaling pathways that are crucial in the progression of the cell cycle as previously described in our group (Cabral et al. 2013; Sanchez and Moreno [Bibr b25][Bibr b26]). Thus, [^3^H]AA release, metabolism by both COXs, and the interaction of PGE_2_ with EP_1_ and EP_4_ receptors are not only important events of acute and chronic inflammation, but are also essential regulators of the proliferation of transformed intestinal epithelial cancer cells induced by LTD_4_.

We recently demonstrated the signaling pathways involved in Caco-2 cell growth induced by PGE_2_ to be ERK, CREB, GSK, and p38, which is consistent with Pham et al. (2008) and Cherukuri et al. (2007). Our results also indicate that LTD_4_ also induces ERK and CREB phosphorylation and that both signaling pathways were completely blocked when COX was inhibited. This therefore indicates that the activation of both ERK and CREB could be attributed to PGE_2_ synthesis induced by LTD_4_. Furthermore, while Caco-2 cells have a high basal β-catenin dephosphorylation level, we found that LTD_4_ induces an additional dephosphorylation, which is consistent with research by Öhd et al. (2000). A previous study on Caco-2 cells in our laboratory revealed that this signaling pathway was not activated by a mitogenic factor such as PGE_2_. Thus, since the mitogenic effect of LTD_4_ is completely PGE_2_-dependent, it is unlikely that the activation of the β-catenin pathway by LTD_4_ is directly related to cell proliferation, and so as reported by Salim et al. (2014), the dephosphorylation of β-catenin induced by LTD_4_ could be linked to the migration of colon cancer cells.

In conclusion, the effects of LTD_4_ appear to occur through the increased expression and activation of COX-2, the production of PGE_2_, and the interaction of PGE_2_ with its cell-surface receptors. These findings will help improve our understanding of how inflammatory mediators can affect the survival and dissemination of intestinal carcinoma cells.
